# Physical cognition: birds learn the structural efficacy of nest material

**DOI:** 10.1098/rspb.2013.3225

**Published:** 2014-06-07

**Authors:** Ida E. Bailey, Kate V. Morgan, Marion Bertin, Simone L. Meddle, Susan D. Healy

**Affiliations:** 1 School of Biology, University of St Andrews, Harold Mitchell Building, St Andrews, Fife KY16 9TH, UK; 2 Roslin Institute, The Royal (Dick) School of Veterinary Studies, University of Edinburgh, Easter Bush EH25 9RG, UK

**Keywords:** bird, cognition, construction, learning, structural properties, nest building

## Abstract

It is generally assumed that birds’ choice of structurally suitable materials for nest building is genetically predetermined. Here, we tested that assumption by investigating whether experience affected male zebra finches’ (*Taeniopygia guttata*) choice of nest material. After a short period of building with relatively flexible string, birds preferred to build with stiffer string while those that had experienced a stiffer string were indifferent to string type. After building a complete nest with either string type, however, all birds increased their preference for stiff string. The stiffer string appeared to be the more effective building material as birds required fewer pieces of stiffer than flexible string to build a roofed nest. For birds that raised chicks successfully, there was no association between the material they used to build their nest and the type they subsequently preferred. Birds’ material preference reflected neither the preference of their father nor of their siblings but juvenile experience of either string type increased their preference for stiffer string. Our results represent two important advances: (i) birds choose nest material based on the structural properties of the material; (ii) nest material preference is not entirely genetically predetermined as both the type and amount of experience influences birds’ choices.

## 1. Introduction

Many animal species collect and use materials from their environment to complete physical tasks, such as building nests, traps, bowers, dams and protective coverings [1]. Success in these tasks will depend on the animal's ability to choose structurally suitable materials. There is compelling evidence, at least for a small proportion of tool-using species, that the ability to choose structurally more appropriate materials becomes refined with experience [2–4]. For most other construction tasks, however, choice of structurally appropriate material has been little studied and is often assumed to be innate [5,6–9], despite an early argument to the contrary [10]. Consequently, the degree to which learning and memory are involved in choosing structurally suitable materials for purposes such as nest building remains largely unknown [11]. As nest construction appears to rely on knowledge of the structural properties of appropriate nest material and is both taxonomically widespread and common, it may be a useful system for investigating the role of cognition in material choice [12].

Birds do appear able to learn at least some aspects of nest material choice: adult zebra finches (*Taeniopygia guttata*) will reverse their colour preference of nest material if they have successfully fledged young from a nest built with nest material of a colour they did not prefer [13]. Choice of material based on its colour tells us little, however, about what birds may learn about the structural properties of materials suitable for building a sound nest. There is some tantalizing evidence that choice of nest material, probably based on its physical properties, changes through experience. For example, young village weaverbirds *Ploceus cucullatus* initially preferred flexible over rigid material and longer over shorter material but did attempt to nest build with materials such as tooth-picks [14]. As the weaverbirds gained nest-building experience, however, they became increasingly discriminating as to the materials with which they would build, to the extent that they rejected artificial materials such as tooth-picks, string and raffia, even when there was no natural alternative available [14]. The weaverbirds’ manipulative skills for cutting and weaving also improved with experience as young male weaverbirds made more mistakes, creating messier and less tightly woven nests than did older, more experienced males [14]. Nest-building experience in lovebirds *Agapornis* spp*.* also improves the efficiency of gathering and transporting of nest material [15] but, to date, little is known about the decision-making processes involved in the selection of structurally suitable materials for nest building.

There are a number of opportunities for birds to learn about the structural properties of nest material: (i) young birds may imprint on the material of the nest in which they hatched and from which they fledged; (ii) birds may be able to assess structural suitability by mandibulating material; (iii) young birds may ‘practise’ building nests; (iv) birds may assess the effort required to build a nest, and (v) birds may associate the success of a nesting attempt with the specific nest materials used [16].

Here, we set out to determine whether learning plays a role in the selection by male nest-building zebra finches of structurally appropriate nest materials. Male zebra finches build nests in a variety of locations using a range of different material. Nests in the wild are usually hollow balls of stiff dry grass stems but they may also be built of fine twigs [17]. Nests may have an entrance tunnel or, alternatively, the birds may skip building the nests’ outer shell almost entirely and nest in a cavity [17]. Zebra finch males will also readily build in captivity using a variety of nest materials. In the following experiments, we provided male zebra finches with two types of string of differing flexibility to see what role, if any, learning played in their choice of nest-building material. We tested three hypotheses.

— *Nest-building experience affects the choice of nest material* (*Experiments 1 and 4*). If zebra finches base their choice of material on prior building experience, material choice is likely to differ among groups with different building experiences.
— *Reproductive success affects the subsequent choice of nesting material* (*Experiment 2*). If zebra finches base their choice of material on reproductive success, they should prefer the material that is associated with reproductive success [18,13].
— *The choice of nest material is based on early-life experiences with nest material* (*Experiment 3*). If early-life experience with nest material affects choice, we would expect to see that this would explain the choice of material used to build the first nest.

## 2. Material and methods

### (a) Experiment 1: effect of building experience

Adult zebra finches were housed in 24 male : female pairs for 6–33 days (mean = 12.83 ± 2.04 s.e. days) prior to the start of the experiment and allowed to form pair bonds. Birds were at least eight months old and had never bred. They were obtained from The University of Glasgow and a pet shop and were all raised following standard breeding protocols. In St Andrews, the pairs were housed in wooden cages that had wire mesh fronts (91 × 31 × 39 cm, length, width, height) on (14 L : 10 D cycle, lights on 08.00 h; ambient temperature 19.6–20.8°C; humidity 53–70%) with ad libitum birdseed, water supplemented with calcium and vitamin D3, cuttlefish bone and oyster shell grit. Birds could hear, but not see, their immediate neighbours but they did have visual and auditory contact with other zebra finches in the room.

On day 7 of the experiment, the birds were provided with a wooden nest-box (11 × 12 × 4.5 cm length, width, height) placed in the centre of either the left- or right-hand half of the cage and hung so that the top was half way up the back wall of the cage. Fifty pieces of either stiff (stiff treatment) or flexible (flexible treatment) string were placed on the cage floor under the nest-box. All string was coloured off-white with a diameter of 2.5 mm and cut into 15 cm lengths. The ‘stiff-treatment’ string was polished cotton and the ‘flexible-treatment’ string was unpolished cotton (both manufactured by James Lever and Sons Ropes and Twines, UK). As a crude comparison of the flexibility of the two materials, a 15 cm length of each string type was hung over a horizontal wire and the distance between the ends measured (distance: stiff-treatment string = 12.5 cm, flexible-treatment string = 11.5 cm). A further 50 pieces of the same string type were provided on day 2. On day 3 or once the males had added all 100 pieces of string to the nest-box, they were given a string-preference test. Although the female may help arrange material in the nest cup, as it is the male zebra finches that choose material for nest construction, we looked only at the males’ material preferences.

### (b) Preference tests

For preference tests in all four experiments, 25 pieces of stiff string were placed in a pile on the cage floor and 25 pieces of flexible string were placed in another. One pile was placed to the right and one to the left of the nest-box. The side of the nest-box on which each string type was placed across treatments was counterbalanced. Once the experimenter left the room nest-building behaviour was digitally recorded using Sony handycams, or SpyCameraCCTV 2.4 GHZ Bird Box cameras. To establish how many pieces birds took to the nest before a stable preference became apparent, in Experiment 1 we recorded at least the first 20 pieces of material the male added to the nest. From these data, we determined that material preference (the proportion of one string type chosen) was stable after 10 choices (Experiment 1: electronic supplementary material) and therefore we used the first 10 choices as a measure of string-type preference in subsequent data analyses. We recorded only the first 10 choices during the preference tests in Experiments 2–4. For all preference analyses, we counted the number of pieces of each type of string the males had chosen out of 10. Analysis was conducted in the statistics package JMP v. 7.0.2 (SAS Institute Inc.).

### (c) Experiment 2: effect of nest-building experience

Experiment 2 began the day after Experiment 1 was completed. The 24 pairs remained in the same housing and under the same husbandry conditions as in Experiment 1 but were also given egg mix (Haith's egg biscuit food) to feed their chicks. The nest-box, which had been removed after their preference test at the end of Experiment 1, was replaced in the cage, and birds were given 100 pieces of string each day up to a maximum of 1300 pieces unless they had not used one or more strings from the day before, or had laid eggs. We photographed nests every day to record changes in nest morphology. We gave pairs 35 days to lay eggs and start incubating and a maximum of 70 days to initiate successful incubation. If they did not initiate incubation, we split the pair up and re-paired both birds with new partners (three pairs). In three instances, the female of a pair died so we re-paired the males from these pairs. Birds that had been re-paired were given 14 days to lay and start incubating a clutch before being classed as having failed (one pair). All birds that were re-paired, three pairs from the stiff-string treatment and three from the flexible-string treatment, repeated Experiment 1 before re-starting Experiment 2 (*n* = 6).

In Experiment 2, zebra finches were provided with either stiff or flexible string to build a complete nest. The material for each pair was chosen on the basis of the string type the male preferred in the preference test at the end of Experiment 1, such that half of the pairs were given their preferred string type and the other half their unpreferred string type. We also counterbalanced for prior experience so that half the birds from each of these groups had prior experience with stiff string, whereas the other half had prior experience with the flexible string. For the three birds that were indifferent after 10 choices (i.e. of 10 pieces they chose four and six or five and five pieces of each string type) at the end of Experiment 1, we used data for their subsequent string choices until they had selected one string type by a ratio of 2 : 1. We then used that choice to allocate them a string type (this took a maximum of 15 choices).

Once the offspring were 30–35 days old, the fledglings and nests were removed. The adults (including those with failed nests *n* = 7) were then given 6 days before being given a preference test.

### (d) Experiment 3: effect of early-life experience on initial string-type preferences

Chicks hatched in Experiment 2 were separated from their parents at independence (30–35 days old depending on when they were first observed to be feeding independently) and housed together in flight cages (140 × 71 × 122 cm, length, width, height, maximum 15 birds per cage) until they could be sexed via their plumage (mean = 39.5 ± 5.11 days). Thirty of the 59 fledglings were male. From then until they were sexually mature (90–100 days of age), the males were grouped in four cages (70 × 71 × 122 cm, length, width, height) and provided with one of the two types of string. Each cage had a different string type: natal nest string-type combination (natal nest/flight cage: flexible/flexible, *n* = 7; flexible/stiff, *n* = 7; stiff/stiff, *n* = 8; stiff/flexible, *n* = 8). The flight cages were constructed of wire mesh with a solid floor and a solid wooden partition to prevent visual contact with males in adjacent cages. The juvenile birds did not see or experience, at any time, any other sort of building material than the one allocated to their treatment group. Two nest-boxes were provided in each flight cage. To provide a song tutor for the development of normal adult song a male/female pair of adult zebra finches were housed in a separate cage in the same room.

One hundred pieces of string were given to the juvenile males when they were first placed in their flight cages, and 100 new pieces of string were added each week unless they had one or more pieces of string left unused on the cage floor (total 600–700 pieces). Juvenile females remained in their flight cages (to which all fledglings had been moved) and were given no experience with building material.

When the juveniles reached maturity, males were paired up with females from the same cohort into the same wooden cages used in Experiments 1 and 2. The pairing of siblings or cousins was avoided. After one week each pair (*n* = 30) was given a nest-box and a choice of stiff and flexible string (25 pieces in each pile of string type) with which to nest build in order to evaluate their initial string-type preference. This evaluation of preference was allowed to run for up to 4 days. If the birds had not taken at least 10 pieces of string to the nest-box during this time, the string and nest-box were removed and replaced 6 days later (three pairs). Two pairs (one from the stiff/stiff treatment and one from the flexible/flexible treatment) failed to take at least 10 pieces of string to the nest-box in this second attempt and so were excluded from the experiment.

### (e) Experiment 4: effect of nest-building experience on first nest string-type preferences

Once all juvenile males’ initial string preferences were evaluated in Experiment 3, the nest-box and all string were removed from the cage and they were left at least 1 day (mean 3.54 ± 1.50 days). The nest-box was then returned and they were given 50 pieces of one of the two string types. Half of the males (*n* = 15) were provided with the string type they had experienced in their flight cages, and the other half the string type they had not experienced in their flight cages. On the subsequent day, they were given another 50 pieces of the same material. On day 3, or once they had added all 100 pieces of string to the nest-box, all of the string they had added to the nest-box was removed and a second preference test was given. A maximum of 4 days was allowed for the birds to add the 100 pieces of string to the nest-box and an additional 2 days allowed for completion of the preference test (four pairs failed to complete the preference test).

## 3. Results

For data, see the electronic supplementary material, S2.

### (a) Experiment 1: effect of building experience

To determine whether the group of six males that repeated Experiment 1 twice made similar choice on both occasions, we compared the percentage of pieces of stiff string they chose in both preference tests. These males’ choices did not differ significantly between the two preference tests (Wilcoxon signed-rank, *W*_4_ = 1.50, *p* = 0.50).

The choice of string was affected by prior building experience. Males that had started building their nests with flexible string chose a lower percentage of flexible string than did males that had started their nest with stiff string (Wilcoxon rank sums test, *Z*_11,13_ = 2.19, *p* = 0.03). Males initially given flexible string preferred stiff to flexible string when tested (mean = 87.69 ± 5.67%, Wilcoxon signed-rank, *W*_12_ = 43.50, *p* < 0.01; preference compared to 50%), whereas birds initially given stiff string were indifferent to string type (mean = 49.09 ± 12.31%, Wilcoxon signed-rank, *W*_10_ = 0.50, *p* = 0.99; preference compared to 50%).

### (b) Experiment 2: effect of nest-building experience

Males that successfully raised chicks did not necessarily prefer the type of string with which they built their nest (Wilcoxon signed-rank, *W*_18_ = 10.50, *p* = 0.63; preference compared to 50%) and their preference for stiff string did not differ from that of males who had failed to raise chicks (means of 88.33 ± 4.37% and 91.67 ± 5.42%, respectively; Wilcoxon rank sums test, *Z*_6,18_ = 0.19, *p* = 0.84). All males in Experiment 2 preferred stiff string (Wilcoxon signed-rank, *W*_23_ = 137.00, *p* < 0.01) and this preference was stronger than it had been in Experiment 1 (means of 89.16 ± 3.51 and 70.00 ± 7.44, respectively; Wilcoxon signed-rank, *W*_23_ = 46.00, *p* < 0.01; preference compared to 50%). The type of string with which they built in Experiment 2 made no clear difference to the strength of that preference (Wilcoxon rank sums test, *Z*_11, 13_ = 1.47, *p* = 0.14).

### (c) String-type preference after multiple nesting experiences

**Figure 1.**
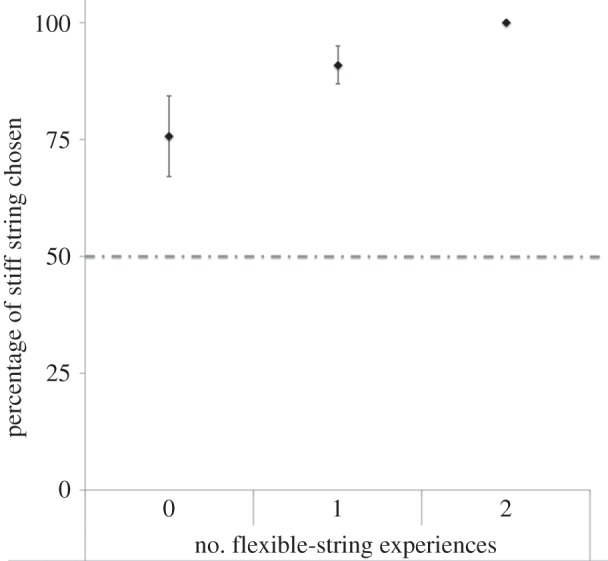
The percentage of stiff string chosen by males that had no (*n* = 7), one (*n* = 10) or two (*n* = 7) experiences of building with flexible string. The data are the means and standard errors for these treatment groups. The dashed line indicates 50%.

To ascertain whether the number of nesting experiences each male had with a string type affected the strength of their preference for the string type with which they built in Experiment 2, the data from Experiment 2 were divided into three groups: (i) males that had experienced flexible string in both experiments, (ii) males that had experienced both stiff and flexible string, and (iii) males that had experienced only stiff string. The more experience the males had of flexible string, the greater their preference for stiff string (Kruskal–Wallis test, *H*_7,10,7_ = 7.42, *p* = 0.02; post-hoc comparisons between groups, flexible only: stiff only, *χ*² = 6.87, *p* < 0.01; flexible only: flexible and stiff, *χ*² = 3.37, *p* = 0.07; stiff only: flexible and stiff, *χ*² = 1.96, *p* = 0.16; figure 1).

### (d) Change in string preference with building experience

**Figure 2.**
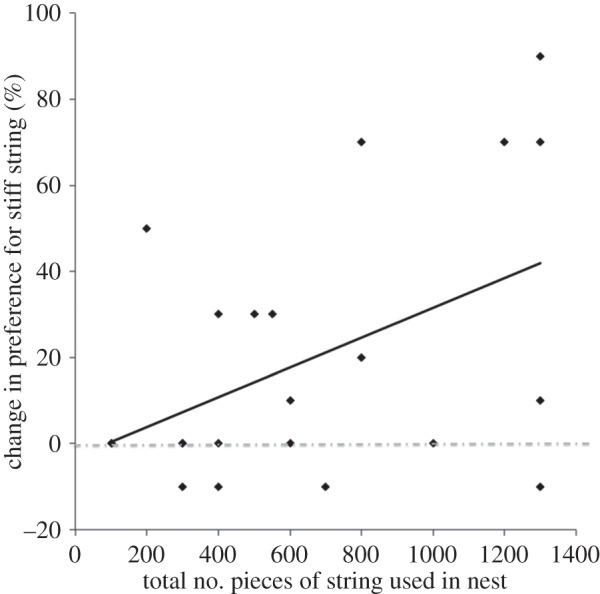
The percentage change in birds’ preference for stiff string from before and after building a complete nest (Experiment 2). Preferences were calculated from the first 10 choices. The data are preferences for each male (*n* = 24). The dashed line indicates no change (0%).

The number of pieces of string the males used to build their nest contributed to the change in preference for string type between the two experiments: the more pieces of either string type males added to their nest during Experiment 2, the more they increased their preference for stiff string (linear regression model, *F*_1,22_ = 6.79, *p* = 0.02; figure 2). The type of string, stiff or flexible, used to build the nest in Experiment 2 was unimportant to both the total number of pieces of string males used to construct their nests (means of 607 ± 107 and 700 ± 152 pieces, respectively; Wilcoxon rank sums test, *Z*_11,13_ = 0.35, *p* = 0.73) and the degree to which they changed their preference for stiff string between preference tests (means of 20.00 ± 8.32 and 18.18 ± 9.79%, respectively; Wilcoxon rank sums test, *Z*_11,13_ = 0.20, *p* = 0.84).

### (e) Nest morphology

Some of our male zebra finches used their nest material to construct a roof on their nest, much as wild zebra finches often do. Therefore, we investigated whether the string type with which the males built their nest affected the likelihood of them building a nest with a string roof: it did not (*χ*²-test, *χ*² = 0.56, *p* = 0.46; *n* = 14).

To determine how readily nests with a roof were built, we compared the number of pieces of string used before a roof first appeared. Although the number of males that built a roof on their nest did not differ depending on the string type, the number of pieces used to achieve a roof did. Males that built a nest with a flexible string roof required many more pieces to achieve this than did males that built a roof with stiff string (pieces of string used to construct a roof: stiff string = mean 469 ± 63, flexible string = mean 800 ± 129, *n* = 6 and 8, respectively; Wilcoxon rank sums test, *Z*_6,8_ = 1.95, *p* = 0.05).

Another strategy that the birds used to acquire a nest with a roof was to build a tower up to the roof of the cage (*n* = 7). Although the string type did not affect the building of a tower nest (three towers were built with flexible string and four with stiff string), tower nests required more string to make than did string-roofed nests (string-roof nests: *n* = 14, mean number of pieces of string = 590, tower nest: *n* = 7, mean number of pieces of string = 986; liner mixed model fitted using restricted maximum-likelihood approach, with bird as a random factor and nest morphology and string type as a main effects; adjusted *r*^2^ = 0.09; nest morphology, *F*_1,13_ = 12.59, *p* < 0.01, all nests built with flexible string tended to contain more string than did nests built of stiff string but this was not significant, *F*_1,7_ = 4.09, *p* = 0.08).

### (f) Experiment 3: effect of early-life nest experience on initial string-type preferences

Regardless of early-life experience with the different string types, juvenile males preferred stiff string above 50% (mean = 83.57 ± 4.25%; Wilcoxon signed-rank, *W*_28_ = 165.00, *p* < 0.01). Furthermore, males raised in stiff-string nests did not differ in their later string-type preference from those males raised in flexible string-nests (means = 89.33 ± 3.71 and 76.92 ± 8.27%, respectively; Wilcoxon rank sums test, *Z*_13,15_ = 0.82, *p* = 0.41). Males that experienced stiff string in their flight cage did not prefer stiff string more or less than did males that experienced flexible string in their flight cage (means = 89.23 ± 6.68 and 78.67 ± 6.16%, respectively; Wilcoxon rank sums test, *Z*_13,15_ = 0.53, *p* = 0.59).

### (g) Nest-building string preference: fathers and sons

To test whether sons shared string-type preferences with their fathers, the fathers were ranked according to their preferences in Experiment 1. For each of the treatments in Experiment 1, males with scores in the top 50% for preference for stiff string were ranked (i) and those with scores in the bottom 50% (ii). Juveniles whose fathers had a stronger tendency to prefer stiff string were no more likely to prefer stiff string than were males whose fathers preferred stiff string less (means = 82.14 ± 6.81 and 85.00 ± 6.86%, respectively; Wilcoxon rank sums test, *Z*_14,14_ = 0.58, *p* = 0.56).

Siblings also did not tend to have the same preferences for string type. The difference between siblings preference was calculated and the variance of this dataset compared (VAR = 424.73) to the variance among the string choices of the cohort (VAR = 425.67). If the brothers had all chosen similarly, we would have expected the variance among the differences between sibling choices to be lower than among choices overall. However, the variances of these two datasets were not significantly different (*F*-test, *F*_28,25_ = 0.99, *p* = 0.49).

### (h) Experiment 4: effect of nest-building experience on first nest string-type preferences

Juvenile males that built with 100 pieces of flexible string preferred stiff string more strongly compared with males that built with 100 pieces of stiff string (means = 91.54 ± 5.19 and 76.92 ± 6.32%, respectively; Wilcoxon rank sums test, *Z*_13,13_ = 2.39, *p* = 0.02).

## 4. Discussion

Popular belief would have it that birds’ choice of structurally appropriate nest material is genetically predetermined [5–9]. We have found, however, that as a result of their building experience, male zebra finches learned to choose stiffer string to build their nests and to avoid building with the more flexible string type. The preference for material type shown by the juvenile males may be influenced by their early-life experiences but we found no evidence that variation in preference prior to building experience was consistent within families.

Building experience by zebra finches lead to their learning about the structural properties of the different string types and, although we do not know what constitutes a ‘good’ nest for a zebra finch, it seems likely that their preference reflected the suitability of the materials for the construction of their nest. Indeed, the stiffer string appeared to be a more appropriate material with which to build, as many fewer pieces were used to build a nest with a roof. Furthermore, the experience of nest building with just 100 pieces of string, half the minimum number required to make a roofed nest, was enough to affect their string choice. In addition, the degree to which the birds changed their preference for stiff string was related to the total number of pieces of string they had added to their nest.

In summary, the more nest-building experience, the more the birds favoured the stiff string. So, although the experience of building with flexible string led to a preference for stiff string sooner, building with stiff string also eventually led to a preference for that string type. Although it is possible that the birds might have used other differences between the string types that were not apparent to us, such as colour or odour, we think this unlikely as those sources of variation would not have led to the experience-dependent effects we observed.

The morphology of the nests the males built was variable, not apparently converging on a similar design as might be predicted from stereotyped behaviour [12]. For example, birds that built a nest with a roof used one of two strategies to achieve that roof, either using the string to construct the roof or building the nest up to just below the cage roof (in some cases, this meant a nest reaching 39 cm above the cage floor). Within-individual and within-species variation in nest morphology and construction has also been observed in weaverbirds (*Ploceus velatus)* in the wild [19,20]. In neither case do we suppose such variability in nest morphology requires ‘higher cognitive’ abilities [12], as is sometimes claimed when a lack of stereotypical sequences are observed in tool manufacture [21]. How substantial the contribution to this variation from experience-dependent sources, for example, dexterity, building experience or social learning, is not yet clear. Further research will be required to differentiate among these possibilities.

We had expected that with prior reproductive success, birds might prefer the type of string with which they built that successful nest [13] but this was not the case. Males that successfully raised chicks in nests constructed from flexible string later preferred to build with stiff string as much as those birds that had raised chicks successfully in nests made from stiff string. From our data, it seems possible that nest builders based their choice of material on the optimum effort required to successfully build a sufficient nest rather than relying on reproductive success itself. In the wild, nest building with fewer pieces would mean fewer trips to collect material entailing less energetic expenditure on acquisition of material as well as less effort in the building itself. This might also lower predation risk. Finally, building a nest with fewer pieces of material should take less time and lead to females laying their eggs sooner.

Prior to building their first nest and irrespective of their experience, juvenile males preferred string that was stiff rather than flexible. This may mean that the juvenile males had an innate preference for stiffer material or, alternatively, that they had sufficient experience with either type of string such that they preferred the stiff string. Given that in Experiment 3 we were not able to examine preference prior to putting the birds into free-flight cages (the males were too young to test at that time), it is not possible to differentiate between these two explanations as yet.

In conclusion, our results show that male zebra finches, based on their experience with nest-building materials, select the material that is most suitable for building. We found no unambiguous support for a heritable component in these decisions. Learning about nesting materials may then be considerably more important to nest construction in many species than has previously been considered [5–9]. If so, nest construction may be a useful study system for better understanding what information animals can and do use to choose suitable materials for completing physical tasks.

## Notes

All experiments were conducted in accordance with ethical review procedures at the University of St Andrews and the UK Animals (Scientific Procedures) Act 1986 and associated guidelines. Efforts were made to minimize the numbers of animals used.
